# Cyclic Strain Alters the Expression and Release of Angiogenic Factors by Human Tendon Cells

**DOI:** 10.1371/journal.pone.0097356

**Published:** 2014-05-13

**Authors:** Rouhollah Mousavizadeh, Shahram Khosravi, Hayedeh Behzad, Robert G. McCormack, Vincent Duronio, Alex Scott

**Affiliations:** 1 Department of Medicine, University of British Columbia, Vancouver, Canada; 2 Department of Physical Therapy, University of British Columbia, Centre for Hip Health and Mobility, Vancouver Coastal Health Research Institute, Vancouver, Canada; 3 Department of Dermatology and Skin Science, University of British Columbia, Vancouver, Canada; 4 Department of Orthopedic Surgery, University of British Columbia, Vancouver, Canada; University of Pittsburgh, United States of America

## Abstract

Angiogenesis is associated with the tissue changes underlying chronic overuse tendinopathy. We hypothesized that repetitive, cyclic loading of human tendon cells would lead to increased expression and activity of angiogenic factors. We subjected isolated human tendon cells to overuse tensile loading using an *in vitro* model (1 Hz, 10% equibiaxial strain). We found that mechanically stimulated human tendon cells released factors that promoted *in vitro* proliferation and tube formation by human umbilical vein endothelial cells (HUVEC). In response to cyclic strain, there was a transient increase in the expression of several angiogenic genes including ANGPTL4, FGF-2, COX-2, SPHK1, TGF-alpha, VEGF-A and VEGF-C, with no change in anti-angiogenic genes (BAI1, SERPINF1, THBS1 and 2, TIMP1-3). Cyclic strain also resulted in the extracellular release of ANGPTL4 protein by tendon cells. Our study is the first report demonstrating the induction of ANGPTL4 mRNA and release of ANGPTL4 protein in response to cyclic strain. Tenocytes may contribute to the upregulation of angiogenesis during the development of overuse tendinopathy.

## Introduction

Tendinopathy is a common overuse injury, prevalent among both athletes and workers [1]. The disorder is often considered a mechanically driven pathology, as repetitive or forceful loading of tendons are well established risk factors [2]. Repetitive strain and shear are thought to induce matrix degeneration in tendon tissue, making the tissue susceptible to damage and eventually to overuse injury [3]. However, the mechanisms which precede the development of symptomatic injury have not been fully described but are felt to be multifactorial, with repetitive strain being an important risk factor [4], [5].

There is evidence of extensive new blood vessel growth in most types of tendinopathy, including Achilles, patellar and lateral epicondyle tendinopathies, as well as the rotator cuff. Histopathological examination has revealed increased numbers of vessels within and around painful tendons [6], [7]. It has been reported that sites of subjectively defined pain, clinically palpated tenderness, tendon thickness and increased colour Doppler signal are anatomically associated, indicating a possible association between pain and neurovascular changes resulting from tendon overuse [1], [8]. However, it must be acknowledged that the colour Doppler signal typically associated with tendinopathy may represent not only angiogenesis, but increased blood flow in vessels which are already present. Angiogenesis may be accompanied by neurogenesis, i.e, nerves may be proliferating along with neovessels in mechanically loaded tendon tissue increasing the level of substance P and other pain-producing substances in tendon; this histological change could lead to the transition to a symptomatic phase in tendinopathy [9].

Tenocytes comprise the main cell population (90%–95%) in tendon tissue, and may be defined as scleraxis-expressing fibroblasts residing within the extracellular matrix of the tendon, and playing a key role in tendon development, adaption and the response to mechanical loading [10]. Tenocytes produce a variety of endogenous cytokines and growth factors which exert both autocrine and paracrine effects [11]. Some in vitro studies have shown that repetitive mechanical loading of tendon cells results in an elevated production of soluble factors which are sometimes characterized as inflammatory, catabolic, or anabolic (e.g. PGE_2_, TGFβ) [12], [13], [14]. Several studies have suggested that such changes in gene expression induced by repetitive loading of tenocytes could lead to tendinopathy [4].

In this study, we investigated the expression and activity of angiogenic factors released by cyclically strained, scleraxis-expressing cells derived from human tendon tissue.

## Materials and Methods

### Cell Culture

Primary human tendon cells were isolated from healthy hamstring (semitendinosis) tendons (excess anterior cruciate ligament autograft material) of male and female patients (n = 4, mean age 25.75 with SEM±5.75 years). The tendon biopsies were minced into 3–5 mm pieces and digested by 1.5 mg/ml Collagenase D (Roche Applied Science, Switzerland, #11088866001) for 20 minutes in a shaker incubator (200 rpm) at 37°C followed by incubation with 0.25% trypsin (TrypLE, Life Technologies, USA, #A1217702) for 3 minutes. After washing with PBS, the digested tissues were cultured in high glucose Dulbecco’s modified Eagle’s medium (DMEM) supplemented with 10% fetal bovine serum, 2 mm L-glutamine, 100 units/ml penicillin, and 100 µg/ml streptomycin in a humidified incubator containing 5% CO_2_ at 37°C. After tendon cell adherence to the tissue culture plates, the cultured cells (at 70% confluence) were subcultured 1∶3 up to five passages to obtain adequate cells.

### Ethics

Because our aim was to examine the potential etiological events of tendinopathy resulting primarily from tensile overload, we elected to use tendon cells from normal (healthy) donors, which necessitated the use of orthopaedic autograft material (semitendinosis tendon). This excess tendon material would otherwise have been discarded. The study was reviewed and approved by the UBC Clinical Research Ethics Board, and each patient provided written informed consent. The primary HUVEC cells were isolated from normal umbilical cords under a UBC approved human ethics certificate.

### Cell Characterization

After each passage, the expression levels of aggrecan, COL1A2, decorin, scleraxis, tenomodulin and nucleostemin were quantified using qPCR in order to characterize the tendon cells’ phenotype. Total RNA was extracted from tendon cells using the RNeasy Plus Universal Mini kit (Qiagen, Germany, #73404) and reverse transcribed to cDNA with a High Capacity cDNA Reverse Transcription Kit (Applied Biosystems, USA, #4368814). The primer sequences were designed and tested using NCBI/Primer-BLAST and GeneRunner (version 3.05) and synthesized by Life Technologies (Table S1). The gene expression levels were determined by FastStart Universal SYBR Green Master mix (Roche Applied Science, USA, #04913914001) using GAPDH as internal control (Figure S1). Based on the increased expression of COL1A2, scleraxis and tenomodulin along with no increased expression of nucleostemin (associated with pluripotent tendon-derived progenitor cells), the cultured tendon cells from passages 3 to 5 were selected for experiments.

### Mechanical Loading

The isolated human tendon cells were seeded on 6 well BioFlex Culture Plates coated with collagen type I (Flexcell International Corporation, USA, #BF-3001C) with a density of 1.2×10^5^ cells per well with high glucose DMEM supplemented with 10% fetal bovine serum, 2 mm L-glutamine, 100 units/ml penicillin, and 100 µg/ml streptomycin. By using FX-4000 T FlexLink Starin Unit (Flexcell International Corporation, USA), isolated tendon cells were exposed to equibiaxial (radial and circumferential) cyclic strain (1 Hz frequency, 10% strain) for up to 24 hours. This strain level has previously been shown to be well tolerated by human tendon cells [15].

### Flow Cytometry

The viability of tendon cells after applied cyclic strain was assessed with propidium iodide staining, as previously described [16]. The strained and unstrained tendon cells were trypsinized and centrifuged along with their supernatant in individual tubes. The cell pellets were washed in PBS and fixed with ice cold 70% ethanol for 1 hour. Then the fixed cells were stained with propidium iodide staining solution [1 ml PBS (Ca^+2^, Mg^+2^ free, 0.1% glucose), 10 microliters Rnase A 100 mg/ml and 5 microliters propidium iodide 10 mg/ml] and incubated for minimum 30 minutes. The DNA content in suspended cells was analyzed using the FL3 channel on a flow cytometer, Coulter Epics XL- MCL (Beckman Coulter Inc, USA), and cell death was calculated based on cell populations in the sub G1 phase of the cell cycle.

### HUVEC Proliferation

Conditioned media (DMEM, 5% FBS) of strained or unstrained tendon cells were harvested after 24 hours and stored at −80°C. Primary human umbilical vein endothelial cells (HUVECs, kindly provided by Dr. Aly Karsan and isolated as previously described [17]) were seeded in 96 well plates at a density of 2×10^4^ cells/well and incubated with the conditioned media of strained or unstrained tendon cells for 24 hours. At this time point cells were assessed with the MTS assay using a modified protocol for CellTiter 96 Aqueous MTS reagent powder (Promega, USA, G1111) and PMS (Sigma, USA, #P9625). 20 microliters of MTS/PMS solution were added to the culture media of cultured cells in 96 well plate. After 3 hours’ incubation at 37°C in a humidified 5% CO_2_ incubator, the absorbance at 490 nm was recorded using an Epoch Microplate Spectrophotometer (BioTek, US).

### Tube Formation Assay

In order to evaluate the angiogenic activity of released factors from tendon cells subjected to cyclic strain, a tube formation assay was used (modified from [18]). The conditioned media of the stretched and non-stretched tenocytes were harvested after 24 hours and stored at −80°C. Flat bottom 96 well plates were coated with 50 µl of Matrigel (BD Matrigel Basement Membrane Matrix, USA, #356231). The HUVEC cells were resuspended in the conditioned media of tendon cells which had been strained or unstrained (controls) for 24 hours. Then 100 µl of the resuspended cells (2×10^4^ cells) were added to each well. After 6 hours’ incubation, the tubular networks which form in the matrigel in each well were micrographed using a digital camera (AxioCam ICm 1, Zeiss, Germany) attached to an inverted microscope (Axio Observer.A1, Zeiss, Germany) with 5x objective lens. In some experiments, the tubular networks were stained with Calcein AM (Trevigen, USA,#4892–010-K) and visualized by fluorescent microsopy [18]. Then The TIF format grey-scale images of biological and technical replicates were analyzed with AngioTool software in order to measure the total tube length per field [19].

### Gene Expression Profiling

Total RNA was extracted and converted to cDNA as explained above in the section titled “cell characterization”. The cDNA templates constructed from RNA samples of strained tendon cells for 4 and 12 hours and unstrained cells were used to profile 84 key genes (Table S2) involved in modulating the biological processes of angiogenesis with a Human Angiogenesis RT^2^ Profiler PCR Array (SA Biosciences, USA, #PAHS-024Z). A three-fold change in gene expression was chosen as a cut off for selecting genes for further analysis. Then conventional qPCR was used to confirm and further analyze the expression pattern of stimulated genes after 1, 2, 4, 6, 8, 12 and 24 hours cyclic strain. The qPCR data were represented as fold change compared to unstrained tendon cells. Gene expression changes from samples harvested at 1, 2, and 4 hours cyclic strain were grouped as early response genes, and gene expression changes from samples harvested at 6, 8, 12 and 24 hours strained were grouped as late response genes. We also analyzed the expression of several of the most well-studied angiogenic factors already suspected to play a role in tendons including VEGFs and bFGF. The primer sequences used in conventional qPCR are shown in Table 1.

**Table 1:** Oligonucleotide sequence of primers and amplicon sizes of selected angiogenic genes.

Target gene	Forward primer sequence	Reverse primer sequence	Amplicon size (bp)
**ANGPTL4**	CTCCCGTTAGCCCCTGAGAG	AGGTGCTGCTTCTCCAGGTG	140
**Cox-2**	CAGGGTTGCTGGTGGTAGGA	GCATAAAGCGTTTGCGGTAC	119
**FGF-1**	GAAGTTTAATCTGCCTCCAGGGAAT	CCCCCGTTGCTACAGTAGAG	63
**FGF-2**	CGGGTGCCAGATTAGCGG	GGGTTCACGGATGGGTGT	114
**TGFA**	CCTTGGAGAACAGCACGTC	CACATGCTGGCTTGTCCTC	147
**SPHK1**	CTTCACGCTGATGCTCACTG	GTTCACCACCTCGTGCATC	124
**VEGFA**	CCTCCGAAACCATGAACTTT	CCACTTCGTGATGATTCTGC	132
**VEGFC**	GCCCCAAACCAGTAACAATC	GCTGGCAGGGAACGTCTAAT	109
**GAPDH**	TCTTTTGCGTCGCCAGCCGAG	TGACCAGGCGCCCAATACGAC	100

### Western Immunoblot

Total protein was harvested in lysis buffer (50 mM Tris-Cl, pH 7.7; 1% Triton X-100; 10% glycerol; 100 mM NaCl, 2.5 mM EDTA, 10 mM NaF) [20] supplemented with complete protease inhibitor cocktail (Roche, Germany, #04693124001) and then homogenized by 3 sonication cycles with 25 watts power output at frequency of 23 kHz for 5 seconds on ice and with 10 seconds interval. The homogenates were spun at 13,000 g for 10 min. The protein concentrations of the supernatants were measured using the BCA Protein Assay Kit (Pierce, ThermoScientific, USA, #23225). The protein was heated for 3 min in 5x loading buffer (Fermentas, Lithuania, #R0891). 20 µg of protein from each aliquot was resolved by electrophoresis in a 12% SDS-PAGE gel at a constant voltage of 110 volts. For the detection of secreted ANGPTL4, 20 µg protein of concentrated conditioned media of strained and unstrained tendon cells was used. The strained cells were subjected to 2 hours of cyclic strain, followed by 6 hours incubation without strain and the serum-free conditioned media of tendon cells were harvested and stored at −80°C. The serum free conditioned media of unstrained tendon cells were used as a control. In this experiment we used serum free media in order to avoid clogging during concentrating the conditioned media. The conditioned media was concentrated with YM-3 Centricon membranes (Millipore, USA, #4302) at 7000 g for 4 h at 4°C. The resolved proteins were transferred to a 0.45 µm nitrocellulose membrane (Biorad, Germany, #162-0115) in a clod transfer buffer (25 mM Tris, 192 mM glycine, 20% methanol) at 35 volts for overnight. The membranes were blocked with 5% milk in Tris-buffer saline with 0.5% Tween 20 (TBST), and probed with anti-ANGPTL4 Antibody (Novex, Life Technologies, USA, #710186) in TBS-T overnight at 4°C, followed by 3×10-min TBST washes, followed by Goat Anti-Rabbit IgG, H & L Chain Specific Peroxidase Conjugate (Calbiochem, USA, #401315) in TBST. Immunoreactivity was detected by SuperSignal West Femto Chemiluminescent Substrate (Thermo Scientific, USA, #PI34095). The density of Western blot bands was quantified by Image J and vinculin protein bands were used as the reference protein for data normalization.

### Enzyme-Linked Immunosorbent Assay (ELISA)

The conditioned media of tendon cells cultured in high glucose DMEM supplemented with 10% fetal bovine serum, 2 mM L-glutamine, 100 units/ml penicillin, and 100 µg/ml streptomycin were harvested after 6 hours cyclic strain and stored at −80°C. The conditioned media of unstrained tendon cells strain were used as control. The protein levels of ANGPTL4 in the samples were quantified using a commercial ELISA kit, Human Angiopoietin-like 4 DuoSet (R&D Systems, USA, #DY3485) according to the manufacturer’s protocol.

### Statistical Analysis

All experiments were repeated four times using the isolated tendon cells from separate patients (n = 4). Results of the MTS assay for HUVEC proliferation were examined with Student’s t-test. Descriptive results from Angiogenesis RT^2^ Profiler PCR Array were analyzed using the Web-Based PCR Array Data Analysis on the SABiosciences website in order to identify significantly regulated genes. The qPCR and Western blot (cell lysate) data were analyzed with one way ANOVA followed by Tukey’s multiple comparison test. The data from ELISA and tube formation assays were analyzed using paired t-tests.

## Results

### Applied Cyclic Strain does not Cause Cell Death in Tendon Cells

**Figure 1:**
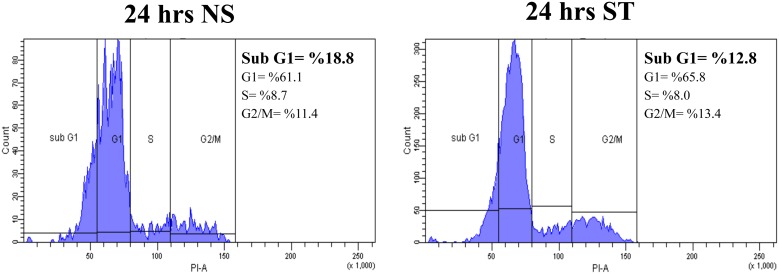
Flow cytometry histograms of tendon cells following propidium iodide staining. The cell death percentage, as represented by cell populations at Sub G1, showed that applied cyclic strain (1 Hz frequency, 10% strain) for 24 hours (b) did not induce cell death compared to unstrained tendon cells (a).

Flow cytometry of human tendon cells showed that the cyclic strain protocol (1 Hz frequency, 10% strain) was well tolerated by tendon cells and did not induce cell death (Fig. 1). This same cyclic strain protocol has previously been shown to lead to increased transcription of other factors known to play a role in tendinopathy, such as Substance P [15].

### Increased Angiogenic Activity of Factors Released by Repetitively Strained Tendon Cells

**Figure 2:**
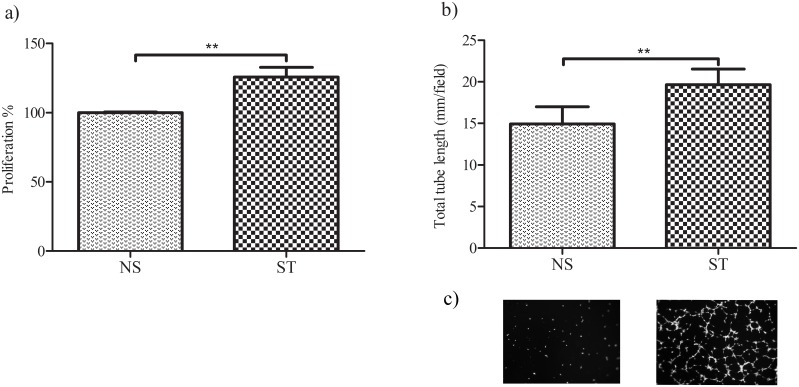
Conditioned media from tendon cells subjected to 24 hours strain (ST) compared to non-strained cells (NS) increase (a) the proliferation of the endothelial cells (HUVEC) and (b) the tubular network formation by HUVECs in Matrigel (a: T test; b: Paired T test; mean ± SE; **, *P*<0.01). (c) The micrographs of calcein AM-labeled tubular network of HUVECs in Matrigel after incubation with conditioned media of non-strained (left) and strained (right) tendon cells.

Incubation of HUVEC cells with the conditioned media from 24 hours strained and unstrained tendon cells showed that cyclic strain resulted in the accumulation of angiogenic factors, since media from loaded tendon cells increased the proliferation of HUVEC cells. In addition, the tube formation analysis showed that the released factors from 24 hours strained tendon cells increased the total length of tubular network of endothelial cells in Matrigel compared to media from unstrained cells (Fig. 2).

### Cyclic Strain Upregulates the Expression of Angiogenic Factors in Tendon Cells

**Figure 3:**
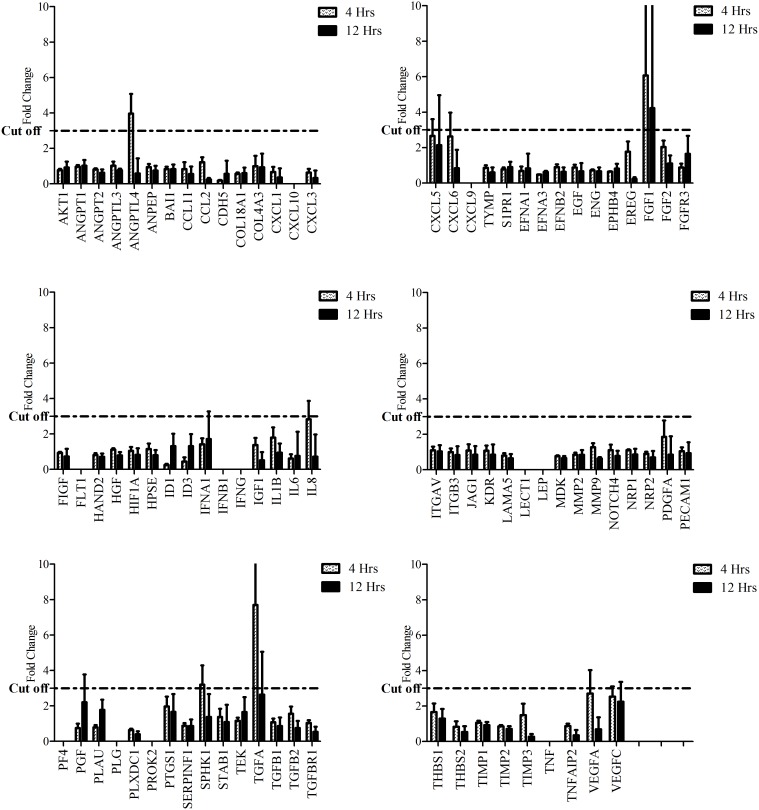
Gene expression array of angiogenic factors in tendon cells after 4 and 12 hours cyclic strain.

**Figure 4:**
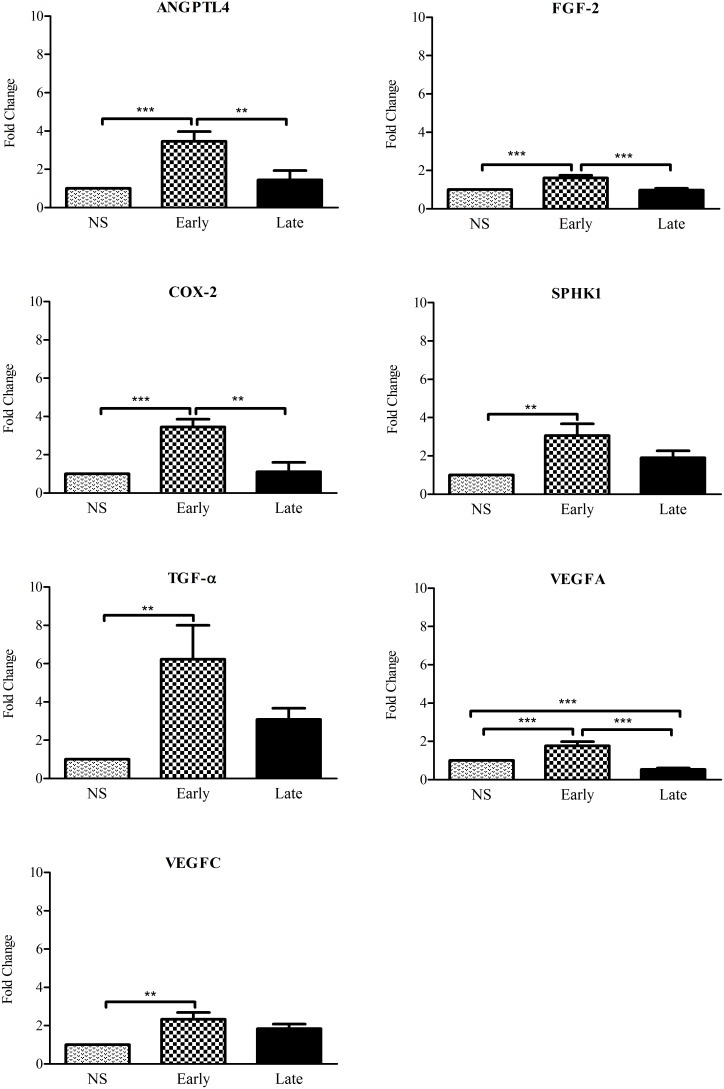
Real-time quantitative PCR on selected genes shows a dynamic response to tensile strain for inducing the expression of most angiogenic factors. (Early response: 1–4 hrs cyclic strain, Late response: 6–24 hrs cyclic strain). (mean ± SE; **, *P*<0.01; ***, *P*<0.001).

Profiling the expression of angiogenic factors by Human Angiogenesis RT^2^ Profiler PCR Array showed that cyclic strain increased the expression of several angiogenic factors, including some that have already been identified as playing a role in tendinopathy, such as VEGF and COX2, along with some novel genes (Fig. 3). Real-time quantitative PCR on selected genes (Fig. 4) showed that cyclic strain within 1–4 hours (early response) transiently upregulated the expression of angiogenic factors including ANGPTL4 (*P*<0.001), FGF-2 (*P*<0.001), COX2 (*P*<0.001), SPHK1 (*P*<0.01, TGF-alpha (*P*<0.01), VEGF-A (*P*<0.001) and VEGF-C (*P*<0.01). In fact, the upregulation of these genes reached peak levels within 1–4 hours of cyclic strain. By extending the time course (longer than 4 hours), the expression of these genes returned to control levels. The early upregulation of mRNA for angiogenic factors in response to cyclic strain was significant for all the above-mentioned genes (Fig. 4). The gene expression array showed no increase in the expression of antiangiogenic factors such as BAI1, SERPINF1, THBS1 and 2, TIMP1-3 (Fig. 3).

### Cyclic Strain Increases the Release of the ANGPT4 Protein

**Figure 5:**
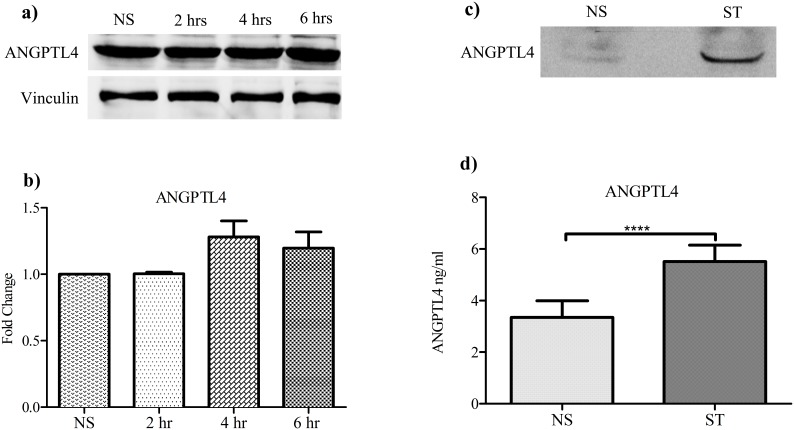
Cyclic loading increases the expression and release of ANGPTL4 in tendon cell culture. (a) Western blot analysis of ANGPTL4 protein in total protein extracts of tendon cells with no strain (NS), 2, 4 and 6 hours strain. (b) Quantitation of the Western blot bands by using vinculin as the loading control. (c) Western blot analysis of ANGPTL4 protein in concentrated serum free conditioned media of tendon cells with no strain compared with 2 hours cyclic strain (ST) followed by 6 hours rest. (d) Increased concentration of ANGPTL4 protein in the conditioned media of tendon cells after 6 hours continuous strain compared with no strain (Paired T test; mean ± SE; ****, *P*<0.0001).

Western blot analysis of cell lysates showed that cyclic strain modulated the level of intracellular ANGPTL4 protein. The extracellular levels of ANGPTL4 in response to strain demonstrated an obvious and statistically significant increase (Fig. 5).

## Discussion

The results of this study build on previous suggestions that angiogenesis may play a role in the pathogenesis of tendon overuse pathology by demonstrating that tendon cells subjected to cyclic strain display marked angiogenic activity. Tendon cells may therefore be partly responsible for the widespread angiogenesis which is known to occur in chronic tendinopathy [21].

In this study we examined the effect of cyclic strain on the expression of a wide array of angiogenic factors. Gene expression analysis demonstrated that cyclic strain of primary tendon cells on tissue culture plates increased the mRNA levels of several angiogenic factors including ANGPTL4, COX-2, bFGF (FGF-2), TGFα, VEGF-A, VEGF-C and SPHK1, while no increased expression of antiangiogenic factors (eg. BAI1, SERPINF1, THBS1 and 2, TIMP1–3) was seen. By extending the time course of cyclic strain, the expression of these same genes was subsequently downregulated after 4 hours of strain. This time course suggests that, in response to repeated bouts of cyclic strain, tendon tissue might be exposed dynamically to repeated, short bursts of angiogenic stimuli which, over time, could lead to an increase in the number of blood vessels in the rotator cuff and associated soft tissues. However, it must be acknowledged that the mechanical loading regimen used in the current study (equibiaxial strain of two dimensional cell culture) is rather different than the mechanical conditions which would be experienced by tenocytes *in vivo*, therefore further study in a suitable laboratory model is required to confirm the physiological relevance of these findings.

There is growing evidence that ANGPTL4 induces a pro-angiogenic response which is independent from the effects of VEGF. Recent studies showed that ANGPTL4 provokes the disruption of vascular junction integrity via integrin α5β1-mediated Rac/PAK signaling and the de-clustering and internalization of VE-caderin and claudin-5 which eventually induce vascular leakiness and permeability [22], [23], [24], [25], [26]. Our study is the first report showing an induction of ANGPTL4 protein in response to mechanical stimulus. Future studies will attempt to unravel the distribution of this protein in tendon tissue from a larger group of patients with chronic tendinopathy. We also plan to examine the potential mechanisms involved in its mechanical regulation.

In summary, this study demonstrates that cyclic strain induces the expression and release of angiogenic factors which can induce endothelial cell proliferation and tube formation. Further work is required to achieve a better understanding of the relationship between repetitive loading and angiogenesis and its potential contribution to the progression of tendon degeneration, particularly with regard to the role of ANGPTL4.

## Supporting Information

Figure S1 **The expression of tendon cell markers and nucleostemin during different passages.** Passages (P) 3–6 were used for experiments.
(TIF)

Table S1 **Oligonucleotide sequence of primers and amplicon sizes of the gene markers for tendon cells.**
(DOCX)

Table S2 **Gene symbols and description of angiogenic factors analyzed by Human Angiogenesis RT^2^ Profiler PCR Array.**
(DOCX)
