# Supplementary figures and images for: Cyclic Strain Alters the Expression and Release of Angiogenic Factors by Human Tendon Cells
Source: PLoS One. 2014 May 13;9(5):e97356. doi: 10.1371/journal.pone.0097356 (PMC4019633; doi:10.1371/journal.pone.0097356)

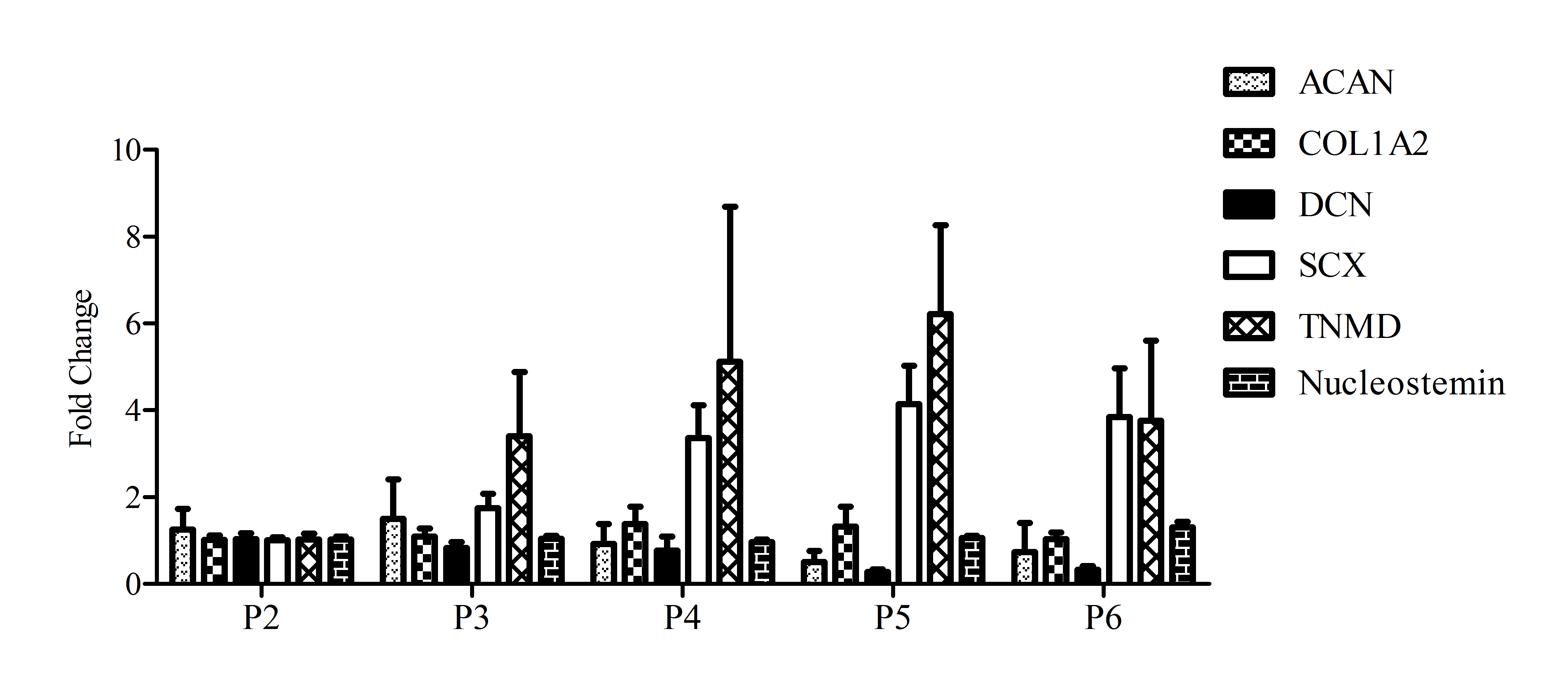

Supplement: Figure S1 — The expression of tendon cell markers and nucleostemin during different passages. Passages (P) 3–6 were used for experiments. (TIF) [file pone.0097356.s001.tif]
