# Supplementary material for: Cyclic Strain Alters the Expression and Release of Angiogenic Factors by Human Tendon Cells
Source: PLoS One. 2014 May 13;9(5):e97356. doi: 10.1371/journal.pone.0097356 (PMC4019633; doi:10.1371/journal.pone.0097356)
Supplement: Table S1 — Oligonucleotide sequence of primers and amplicon sizes of the gene markers for tendon cells. (DOCX) [file pone.0097356.s002.docx]

| Target gene | Forward primer sequence | Reverse primer sequence | Amplicon size (bp) |
| --- | --- | --- | --- |
| Agrecan | GTGTAAAAAGGGCACAGCCAC | ACCAACGATTGCACTGCTCT | 478, 360 |
| COL1A2 | AGTGTCCACGTCCTCAAAAAGA | CAGCAAAGTTCCCACCGAGA | 599 |
| Decorin | GTCACAGAGCAGCACCTACC | TTGTCCAGACCCAAATCAGAACA | 378 |
| Scleraxis | AAGAAAAGCCAGCGCAGAAAGTTC | TCTGCACCTTCTGCCTCAGCAA | 320 * |
| Tenomodulin | GAAGCGGAAATGGCACTGATGA | TGAAGACCCACGAAGTAGATGCCA | 82** |
| Nucleostemin | GGGAAGATAACCAAGCGTGTG | CCTCCAAGAAGTTTCCAAAGG | 98*** |
| GAPDH | TCTTTTGCGTCGCCAGCCGAG | TGACCAGGCGCCCAATACGAC | 100 |
| *Qi J, Dmochowski JM, Banes AN, Tsuzaki M, Bynum D, et al. (2012) Differential expression and cellular localization of novel isoforms of the tendon biomarker tenomodulin. Journal of applied physiology 113: 861-871.  **Bayer ML, Schjerling P, Herchenhan A, Zeltz C, Heinemeier KM, et al. (2014) Release of tensile strain on engineered human tendon tissue disturbs cell adhesions, changes matrix architecture, and induces an inflammatory phenotype. PloS one 9: e86078.  ***Kafienah W, Mistry S, Williams C, Hollander AP (2006) Nucleostemin is a marker of proliferating stromal stem cells in adult human bone marrow. Stem cells 24: 1113-1120. | | | |
