# Supplementary material for: Cyclic Strain Alters the Expression and Release of Angiogenic Factors by Human Tendon Cells
Source: PLoS One. 2014 May 13;9(5):e97356. doi: 10.1371/journal.pone.0097356 (PMC4019633; doi:10.1371/journal.pone.0097356)
Supplement: Table S2 — Gene symbols and description of angiogenic factors analyzed by Human Angiogenesis RT2 Profiler PCR Array. (DOCX) [file pone.0097356.s003.docx]

| **Symbol** | **Description** |
| --- | --- |
| AKT1 | V-akt murine thymoma viral oncogene homolog 1 |
| ANGPT1 | Angiopoietin 1 |
| ANGPT2 | Angiopoietin 2 |
| ANGPTL3 | Angiopoietin-like 3 |
| ANGPTL4 | Angiopoietin-like 4 |
| ANPEP | Alanyl (membrane) aminopeptidase |
| BAI1 | Brain-specific angiogenesis inhibitor 1 |
| CCL11 | Chemokine (C-C motif) ligand 11 |
| CCL2 | Chemokine (C-C motif) ligand 2 |
| CDH5 | Cadherin 5, type 2 (vascular endothelium) |
| COL18A1 | Collagen, type XVIII, alpha 1 |
| COL4A3 | Collagen, type IV, alpha 3 (Goodpasture antigen) |
| CXCL1 | Chemokine (C-X-C motif) ligand 1 (melanoma growth stimulating activity, alpha) |
| CXCL10 | Chemokine (C-X-C motif) ligand 10 |
| CXCL3 | Chemokine (C-X-C motif) ligand 3 |
| CXCL5 | Chemokine (C-X-C motif) ligand 5 |
| CXCL6 | Chemokine (C-X-C motif) ligand 6 (granulocyte chemotactic protein 2) |
| CXCL9 | Chemokine (C-X-C motif) ligand 9 |
| TYMP | Thymidine phosphorylase |
| S1PR1 | Sphingosine-1-phosphate receptor 1 |
| EFNA1 | Ephrin-A1 |
| EFNA3 | Ephrin-A3 |
| EFNB2 | Ephrin-B2 |
| EGF | Epidermal growth factor |
| ENG | Endoglin |
| EPHB4 | EPH receptor B4 |
| EREG | Epiregulin |
| FGF1 | Fibroblast growth factor 1 (acidic) |
| FGF2 | Fibroblast growth factor 2 (basic) |
| FGFR3 | Fibroblast growth factor receptor 3 |
| FIGF | C-fos induced growth factor (vascular endothelial growth factor D) |
| FLT1 | Fms-related tyrosine kinase 1 (vascular endothelial growth factor/vascular permeability factor receptor) |
| HAND2 | Heart and neural crest derivatives expressed 2 |
| HGF | Hepatocyte growth factor (hepapoietin A; scatter factor) |
| HIF1A | Hypoxia inducible factor 1, alpha subunit (basic helix-loop-helix transcription factor) |
| HPSE | Heparanase |
| ID1 | Inhibitor of DNA binding 1, dominant negative helix-loop-helix protein |
| ID3 | Inhibitor of DNA binding 3, dominant negative helix-loop-helix protein |
| IFNA1 | Interferon, alpha 1 |
| IFNB1 | Interferon, beta 1, fibroblast |
| IFNG | Interferon, gamma |
| IGF1 | Insulin-like growth factor 1 (somatomedin C) |
| IL1B | Interleukin 1, beta |
| IL6 | Interleukin 6 (interferon, beta 2) |
| IL8 | Interleukin 8 |
| ITGAV | Integrin, alpha V (vitronectin receptor, alpha polypeptide, antigen CD51) |
| ITGB3 | Integrin, beta 3 (platelet glycoprotein IIIa, antigen CD61) |
| JAG1 | Jagged 1 |
| KDR | Kinase insert domain receptor (a type III receptor tyrosine kinase) |
| LAMA5 | Laminin, alpha 5 |
| LECT1 | Leukocyte cell derived chemotaxin 1 |
| LEP | Leptin |
| MDK | Midkine (neurite growth-promoting factor 2) |
| MMP2 | Matrix metallopeptidase 2 (gelatinase A, 72kDa gelatinase, 72kDa type IV collagenase) |
| MMP9 | Matrix metallopeptidase 9 (gelatinase B, 92kDa gelatinase, 92kDa type IV collagenase) |
| NOTCH4 | Notch 4 |
| NRP1 | Neuropilin 1 |
| NRP2 | Neuropilin 2 |
| PDGFA | Platelet-derived growth factor alpha polypeptide |
| PECAM1 | Platelet/endothelial cell adhesion molecule |
| PF4 | Platelet factor 4 |
| PGF | Placental growth factor |
| PLAU | Plasminogen activator, urokinase |
| PLG | Plasminogen |
| PLXDC1 | Plexin domain containing 1 |
| PROK2 | Prokineticin 2 |
| PTGS1 | Prostaglandin-endoperoxide synthase 1 (prostaglandin G/H synthase and cyclooxygenase) |
| SERPINF1 | Serpin peptidase inhibitor, clade F (alpha-2 antiplasmin, pigment epithelium derived factor), member 1 |
| SPHK1 | Sphingosine kinase 1 |
| STAB1 | Stabilin 1 |
| TEK | TEK tyrosine kinase, endothelial |
| TGFA | Transforming growth factor, alpha |
| TGFB1 | Transforming growth factor, beta 1 |
| TGFB2 | Transforming growth factor, beta 2 |
| TGFBR1 | Transforming growth factor, beta receptor 1 |
| THBS1 | Thrombospondin 1 |
| THBS2 | Thrombospondin 2 |
| TIMP1 | TIMP metallopeptidase inhibitor 1 |
| TIMP2 | TIMP metallopeptidase inhibitor 2 |
| TIMP3 | TIMP metallopeptidase inhibitor 3 |
| TNF | Tumor necrosis factor |
| TNFAIP2 | Tumor necrosis factor, alpha-induced protein 2 |
| VEGFA | Vascular endothelial growth factor A |
| VEGFC | Vascular endothelial growth factor C |
